# Alternative Splicing of CIPK3 Results in Distinct Target Selection to Propagate ABA Signaling in Arabidopsis

**DOI:** 10.3389/fpls.2017.01924

**Published:** 2017-11-24

**Authors:** Sibaji K. Sanyal, Poonam Kanwar, Harsha Samtani, Kanwaljeet Kaur, Saroj K. Jha, Girdhar K. Pandey

**Affiliations:** Department of Plant Molecular Biology, University of Delhi South Campus, New Delhi, India

**Keywords:** abiotic stress, alternative splicing, ABR1, calcium, CBL, CIPK, signal transduction

## Abstract

Calcium (Ca^2+^) signaling is pivotal in transmission of information in the cell. Various Ca^2+^ sensing molecules work to sense and relay the encrypted messages to the intended targets in the cell to maintain this signal transduction. CBL-interacting protein kinases (CIPKs) are crucial components of Ca^2+^ signal transduction during various abiotic stresses. Although there are intron rich CIPKs in the plant genome but very little has been reported about their alternative splicing. Moreover the physiological significance of this event in the Ca^2+^ signaling is still elusive. Therefore in this study, we have selected CIPK3, which has highest number of splice variants amongst Arabidopsis CIPKs. Expression profiling of five splice variants of CIPK3 by qRT-PCR in four *Arabidopsis thaliana* ecotypes revealed preferential transcript accumulation but similar subcellular localization of the variants and interaction with similar CBLs. ABA and drought treatment resulted in the higher accumulation of the alternately spliced transcripts of *CIPK3* in Arabidopsis ecotype *Wassilewkija.* The transcripts of *CIPK3.1* and *CIPK3.4* are relatively more induced compared to other alternative splice variants. Out of four splice variants studied, we found CIPK3.1 and CIPK3.2 showing preference for ABR1, a previously reported interactor of CIPK3. We conclude that the differential expression and choice of downstream partner by CIPK3-splice variants might be one of the mechanisms of Ca^2+^ mediated preferential regulation of ABA and other stress signals.

## Introduction

Calcium (Ca^2+^) is a very important molecule at the center of signal transduction pathway (Sanders et al., 2002; Kudla et al., 2010). It efficiently transduces physiological, stress (abiotic and biotic) and developmental signals (Hepler, 2005; Reddy et al., 2011; Zhu, 2016). Also it integrates quite well with other signaling pathways in plants like ABA and reactive oxygen species (ROS) (Steinhorst and Kudla, 2014; Edel and Kudla, 2016). Being a central messenger, the decoding mechanism of Ca^2+^ is a well-characterized system in plants (DeFalco et al., 2010; Dodd et al., 2010). The evolution of this mechanism from unicellular organisms to multicellular plants is very intriguing. The presence of numerous signal sensors and sensor-decoders makes up a robust and quick signal-response network (Hashimoto and Kudla, 2011). But what makes it interesting is when alternative splicing (AS) generates multiple transcripts to add complexity to the transduction machinery (Filichkin et al., 2015; Wang et al., 2015). AS is an important mechanism for generating diversity of the resulting proteins (Reddy et al., 2013). AS affects the binding property, intracellular localization, enzymatic activity, protein stability and posttranslational modification of a large number of proteins (Wang et al., 2015). In Arabidopsis, mitogen *activated kinase13* (*MPK13*) present an example of AS controlling enzyme activity (Lin et al., 2010). The YUCCA4 splice variants control auxin biosynthesis by being differentially localized in the cell (Kriechbaumer et al., 2012). AS of ZIFL1 transporter generate spliced variants that separately take part in auxin related processes and mediate drought tolerance (Remy et al., 2013). Arabidopsis protein type 2C phosphatase (PP2C) HAB1 also undergoes AS to generate two variants that can control ABA signaling with its downstream interactor OST1 by differentially modulating OST1 kinase activity (Wang et al., 2015).

The Calcineurin B-like proteins (CBL) are efficient Ca^2+^ signal decoders and require CBL-interacting protein kinases (CIPK) to relay the messages across various cellular targets (Luan, 2009; Dodd et al., 2010). The existence of the module, i.e., CBL-CIPK has been proved in both higher and lower plants (Kleist et al., 2014; Pandey et al., 2014; Beckmann et al., 2016). They relay signals that arise due to both biotic and abiotic stresses and plant development. The genome of the model plant Arabidopsis has 10 CBLs and 26 CIPKs (Kolukisaoglu et al., 2004; Batistic et al., 2011). The analysis of genome sequencing results and curated databases of Arabidopsis, cassava and rice has shown AS driven existences of splice variants of both CBLs and CIPKs (Kolukisaoglu et al., 2004; Kanwar et al., 2014; Hu et al., 2015). Interestingly, Arabidopsis CIPK3 contain highest number of the splice variants among all members of CBL and CIPK gene family, which make this an interesting candidate for studying the diversity of splice variants. Besides the splice variants what made CIPK3 an exciting target is its role in integrating ABA and Ca^2+^ signals in Arabidopsis. ABR1, a downstream target of CIPK3, can be directly modulated by both ABA (through Ying-Yang) and Ca^2+^ signaling pathway by CBL9-CIPK3 module (Li et al., 2016; Sanyal et al., 2017). Here we analyzed the CIPK3 splice variants to understand their functional and regulatory role in ABA signaling pathway. The detailed understanding of CIPK3 splice variants will enable us to rebuild a basic working model for the functioning of splice variants of CIPKs.

## Materials and Methods

### Plant Material and Stress Treatment

*Arabidopsis thaliana* Wassilewkija (Ws), Columbia-0 (Col-0), Landsberg *erecta* (L*er*) and Bayreuth (Bay-0) ecotype seedlings were grown on half strength Murashige and Skoog (MS) agar plates vertically in culture room at 22°C under 16/8 h light/dark cycle conditions for 2 weeks and then RNA was extracted from them. These were used for ecotype specific studies of CIPK3 splice variants. Abiotic stress treatments were subjected to Ws plants. For Abscisic acid (ABA) 100 μM of ABA was sprayed on the seedlings and water was sprayed in controls. For salt stress, seedlings were grown in 300 mM NaCl plates for the indicated period of time. For drought stress seedlings were exposed in the laminar airflow for the indicated period of time. For cold treatment seedlings were transferred to the 4°C cold room with standard light regime. For ABA, salt, drought and cold stresses, the seedlings after application of stress were incubated in white light for entire duration of treatment. These treatments were executed according to (Kim et al., 2003; Pandey et al., 2005). The bacterial pathogen *Pseudomonas syringae* pv. *tomato* DC3000 [Pst (DC3000)] was provided by Prof. A. K. Nandi, Jawaharlal Nehru University, India. This was grown on King’s medium agar plates or in liquid medium supplemented with 50 μg ml^-1^ rifampicin and 50 μg ml^-1^ kanamycin at 28°C. 5 × 10^5^ cells from the overnight culture were re-suspended in 10 mM MgCl_2_ and injected in the leaves of 4 week old Col-0 plants with a syringe. 10 mM MgCl_2_ was injected as mock, which served as a control. Leaf samples from both treatments were harvested at the mentioned time points for RNA extraction and expression profiling.

### RNA Extraction and qRT-PCR

RNA was extracted and purified from Arabidopsis tissues according to (Sanyal et al., 2017). For qRT-PCR analysis primers were designed manually after identification of unique sites in the cDNA of each splice variants. The primers used are mentioned in Supplementary Table S2. qRT-PCR reaction was done according to (Singh et al., 2015) using KAPPA SYBR green master mix. The relative expression levels of the splice variants were calculated according to (Wang et al., 2015) and *ACTIN2* was used as endogenous control.

### Localization and Co-localization Studies

Four CIPK3 splice variants (CIPK3.1, CIPK3.2, CIPK3.3 and CIPK3.4) were amplified from drought treated Arabidopsis Ws ecotype and cloned in pENTR^TM^/D-TOPO using Invitrogen kit. The primers used for cloning are mentioned in Supplementary Table S3. The constructs were confirmed by sequencing. They were mobilized to pSITE2CA vector (Chakrabarty et al., 2007). The *Nicotiana benthamiana* transformation and visualization of GFP constructs were done according to (Sanyal et al., 2017). The RFP:ABR1 construct was already mentioned in (Sanyal et al., 2017) and the co-localization methodology is also same as (Sanyal et al., 2017).

### Yeast Two-Hybrid Analysis

All the Arabidopsis CBLs (1–10) cloned in pGBT9.BS were mentioned in (Pandey et al., 2015). ABR1:AD clone was mentioned in (Sanyal et al., 2017). Four CIPK3 splice variants (CIPK3.1, CIPK3.2, CIPK3.3 and CIPK3.4) were cloned in pGAD.GH and pGBT9.BS vector. CIPK3K was amplified from CIPK3.1 variant with respective primers and cloned in pGBT9.BS vector. The primers used for cloning are mentioned in Supplementary Table S3. The constructs were confirmed by sequencing. Yeast two-hybrid (Y2H) assay was performed as described in (Sanyal et al., 2017).

Quantification of β-galactosidase activity was performed in triplicate by using ortho-Nitrophenyl-β- galactopyranoside (ONPG) as the substrate according to (Shi et al., 1999). Briefly cells were grown overnight in –LW media. Next day a secondary culture was inoculated and the cells were allowed to grow till the O.D reached 1 (measured at 600 nm). The cells were then pelleted and washed with Z buffer. These cells were re-suspended in Z buffer and were subjected to three cycles of freeze (liquid nitrogen 1 min) and thaw (water bath 37°C for 3 min). The cells were re-pelleted and the supernatant was collected. Freshly prepared 200 μl of ONPG (4 mg/ml) was added to the supernatant and kept in dark at 30°C for 1 h. 600 μl 1M Na_2_CO_3_ was added to the mixture to stop the reaction. OD was recorded at 420 nm and calculations were performed.

### Purification of Fusion Proteins, Site Directed Mutagenesis and *in Vitro* Protein Kinase Assay

All coding DNA sequences (CDS) were amplified and cloned in pGEX4T-3 unless where mentioned. The CIPK3.1T/D and CBL9 constructs were mentioned in (Sanyal et al., 2017). CIPK3.4WT was used as a template and was used to generate a CIPK3.4TD construct by using the same protocol and primers mentioned in (Sanyal et al., 2017). All constructs were confirmed by sequencing. The constructs were transformed in *Escherichia coli* BL21 cells for recombinant protein production. The proteins expression, purification and *in vitro* phosphorylation assay was done according to (Sanyal et al., 2017).

## Results

### Arabidopsis CIPK3 Has Five Splice Variants and Four Exhibit Similar Localization Pattern

**FIGURE 1:**
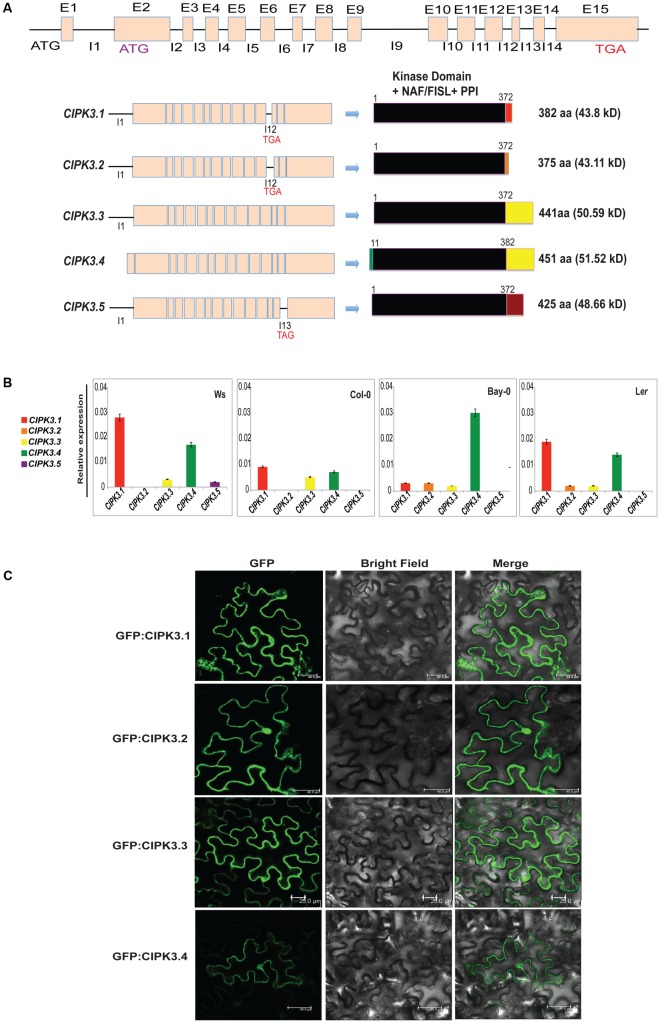
Characteristics of alternative spliced variants of CIPK3. **(A)** Schematic diagram of the organization of CIPK3 locus, transcripts generated from it and the probable proteins coded from these sequences. Black lines indicate the introns and boxes indicate the exons. All the CIPK3 proteins contain all the important motifs (kinase domain, NAF/FISL and PPI) indicated by the black boxes. Colored boxes mark the different regions in the proteins. Alignment of the protein sequences is provided in **Supplementary Figure S1** along with the major motifs of CIPK3 marked by boxes. **(B)** Expression profiles of the CIPK3 splice variants in different ecotypes under normal condition. qRT-PCR was performed for CIPK3 splice variants on Ws, Bay-0, Col-0 and L*er* ecotypes. **(C)** Subcellular localization of CIPK3.1, CIPK3.2, CIPK3.3 and CIPK3.4 proteins in the epidermal peel cells of *Nicotiana benthamiana*. All the constructs showed cytoplasmic and nucleoplasmic localization when transiently expressed in *N. benthamiana* epidermal cells.

Earlier it was reported that the CIPK3 locus generates 3 splice variants (Kolukisaoglu et al., 2004). However, current genome annotation on TAIR^1^ and ACEVIEW^2^ predicted the existence of 15 exons in CIPK3 gene and generation of probable 5 alternate transcripts (CIPK3.1, CIPK3.2, CIPK3.3, CIPK3.4 and CIPK3.5). CIPK3.4 (AT2G26980.4) is the full-length transcript followed by CIPK3.3 (AT2G26980.3) that arises from an alternative start site in the second exon. The predicted start codons of these two transcripts are in frame; so their coding sequences are identical downstream of the CIPK3.3 ATG. This ATG is also the start site of the other three smaller transcripts CIPK3.1 (AT2G26980.1), CIPK3.2 (AT2G26980.2), and CIPK3.5 (AT2G26980.5) that are generated due to intron retention. CIPK3.1 and CIPK3.2 retain intron 12 and generate proteins of 382 aa and 375 aa residues, respectively. The CIPK3.5 variant retain intron 13 and generate 425 aa residues protein. The CIPK3.4 transcript has extended N-terminus of 30 base pairs making it the largest protein with 451 aa residues. CIPK3.3 is the second largest protein with 441 aa residues (**Figure 1A** and **Supplementary Figure S1**). Previous reports where these splice variants were used are mentioned in Supplementary Table S1. Nevertheless to examine the five transcripts in parallel, we performed quantitative real time-PCR (qRT-PCR). As most of the cDNA sequence of CIPK3 splice variants had sequence similarity and we had to identify the unique portions in the transcript to design specific primers. Specific portions were selected for primer designing based on the exclusivity of the sequence (**Supplementary Figure S2**). These sequences were amplified and analyzed by agarose gel electrophoresis (**Supplementary Figure S2**), and subsequently were cloned in primary cloning vector (pJET1.2) and sequenced to confirm the unique variants. qRT-PCR was done on the cDNA made from the RNA of four different Arabidopsis ecotype (Ws, Col-0, L*er* and Bay-0) that were not subjected to any stress. As shown in **Figure 1B**, the expression of *CIPK3.5* transcripts was detected at low level in Ws ecotype. *CIPK3.1*, *CIPK3.3* and *CIPK3.4* were more abundant variants followed by *CIPK3.2*, which had a slightly lower presence.

We then investigated the subcellular localization of the splice variants as often splicing is reported to generate alternately localized protein (Kriechbaumer et al., 2012; Wang et al., 2015). Although CIPKs do not have any yet reported localization motifs in their sequence, we speculated that the smaller proteins (CIPK3.1 and CIPK3.2) might have different subcellular localization. Moreover database search [TargetP 1.1^3^ and WoLF PSORT^4^] with the CIPK3 splice variant sequence pointed to a differential targeting of CIPK3.4 (for which nucleus was predicted) than the other variants (mitochondrial was predicted for the rest). We tested the localization by expressing all the variants downstream of GFP in *Nicotiana benthamiana*. As expected the full-length proteins GFP:CIPK3.4 and GFP:CIPK3.3 localized to the both nucleus and cytoplasm validating localization pattern shown by other CIPKs (**Figure 1C**) (Batistic et al., 2010). But to our surprise, the other two smaller variants (GFP:CIPK3.1 and GFP:CIPK3.2) also showed similar localization (**Figure 1C**). This suggested that the splicing does not alter the localization of CIPK3 variants in the cell.

### Interaction and Phosphorylation Pattern of CIPK3 Splice Variants with Their Upstream CBL Interactors

**FIGURE 2:**
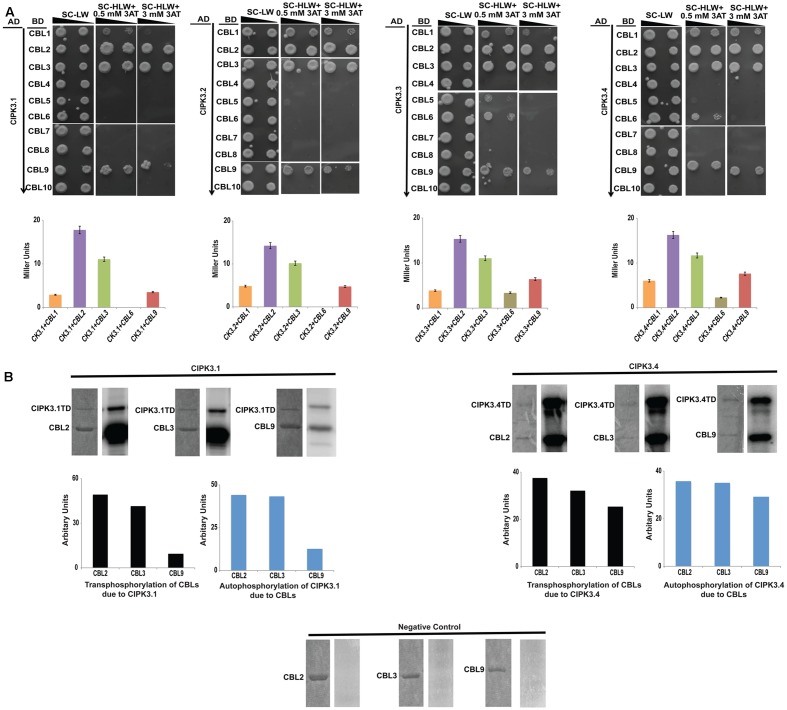
CIPK3 demonstrate similar preference of CBLs. **(A)** Dilution series of yeast AH109 strains transformed with AD-CIPK3 (indicated which splice variant used) and BD-CBLs (CBL1-CBL10). Yeast two-hybrid analysis identified CBL2 and CBL3 as the major interactors of CIPK3 splice variants followed by CBL9 and CBL1. CBL6 showed interaction with only CIPK3.3 and CIPK3.4. The decreasing cell densities in the dilution series are illustrated by narrowing triangles. Yeast was grown on SD-LW medium (first column), SD-HLW + 0.5 mM 3AT medium (second column) and SD-HLW + 3 mM 3AT medium (third column). Results of negative control are depicted in **Supplementary Figure S3A**. Quantitative analysis of β-galactosidase activity of yeast colonies containing CIPK3/CBLs complex. Data shown are in Miller Units. **(B)** *In vitro* kinase activities of CIPK3 splice variants. The GST-CIPK3.1TD and GST-CIPK3.4TD proteins are able to phosphorylate GST-CBL2, GST-CBL3 and GST-CBL9. The CBLs do not have any inherent phosphorylation activity (negative control). Coomassie gels are followed by autoradiogram. The extent of transphosphorylation of the CBL2, CBL3 and CBL9; and autophosphorylation of CIPK3.1 and CIPK3.4 were quantified using ImageJ software.

Alternative splicing could provide selective advantage for choosing upstream regulators (Lin et al., 2010). So we investigated the effect of AS on the regulation of the variants by performing Y2H assay with all the 10 Arabidopsis CBLs. The information on the clones is mentioned in the materials and methods section. The results obtained by spot dilution assay (**Figure 2A**) could be divided into three categories- (a) CBL2 and CBL3 were the strongest interactors for all the splice variants, (b) this was followed by CBL9 and then CBL1 that show weaker interaction strength than the first group, and (c) CBL4, CBL5, CBL7, CBL8 and CBL10 are non-interactors. The first two group members were preferred by all the variants and only CBL6 displayed a very weak interaction with CIPK3.3 and CIPK3.4 (designated by their growth at a very low 3-AT concentration), indicating that majorly same CBLs were preferred by the variants. The interaction strength was also validated by ONPG based β-galactosidase assay (**Figure 2A**).

CIPK3 is a member of the Ser/Thr kinase family and possess phopsotransferase activity. Therefore, we decided to investigate phosphotransferase activity by using CBLs as substrates with two CIPK3 variants, CIPK3.1 and CIPK3.4. Our choice was based on the fact that CIPK3.1, which is structurally similar to CIPK3.2 differing in only the last three amino acids at CIPK3.2’s tail, and CIPK3.4, structurally similar to CIPK3.3 differing in only first 10 amino acids present at the N-terminal of CIPK3.4, would be representative of the CIPK3.2 and CIPK3.3 respectively. We choose CBL2, CBL3 and CBL9 as the substrates since their interaction to CIPK3 has been reported earlier (Pandey et al., 2008; Tang et al., 2015). No phosphorylation was observed when the CBLs were incubated without kinase excluding any chance of non-specific ATP binding to them (negative control in **Figure 2B**). Both the variants were able to phosphorylate CBL2, CBL3 and CBL9 indicating that splicing has not resulted into any selective advantage or disadvantage to the variants, both in terms of interaction to CBLs and phosphorylating them (**Figure 2B**). When we quantified the transphosphorylation of CBLs by CIPK3.1 and CIPK3.4, we observed that both variants preferentially phosphorylated CBL2 and CBL3 more than CBL9. Similarly quantification of autophosphorylation of the CIPK3.1 and CIPK3.4 variants in presence of CBL2 and CBL3 showed enhancement compared to CBL9. These two results together pointed to a fact that probably CBL2 and CBL3 are the better substrates of CIPK3 than CBL9 and they can enhance the *in vitro* activity of CIPK3 better than CBL9.

### Stress Influences the Transcript Abundance of CIPK3 Splice Variants

**FIGURE 3:**
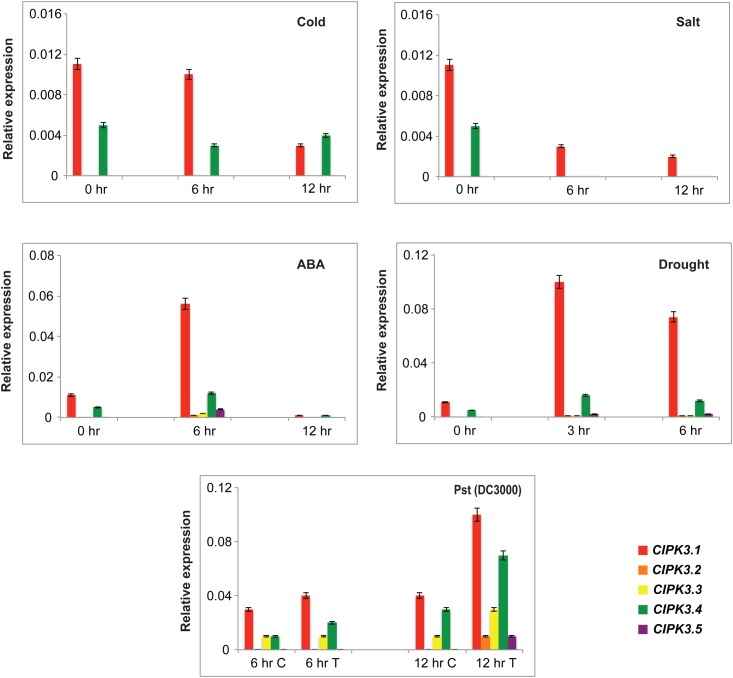
Expression profiles of the CIPK3 splice variants under different stress conditions. qRT-PCR was performed for CIPK3 splice variants. For abiotic stress treatment Ws seedlings were stressed for the indicated time points. The treatment methodology is mentioned in materials and methods section. For biotic stress treatment Col-0 ecotype was used. The plants were treated with either 10 mM MgCl_2_ (control or C) or Pst (DC3000) (Treated or T). All expression were normalized with Arabidopsis *ACTIN2*. Red bars indicate CIPK3.1, orange-CIPK 3.2, yellow-CIPK3.3, green-CIPK3.4 and dark purple-CIPK3.5.

CIPK3 is induced under ABA and different abiotic stresses (Kim et al., 2003). Therefore we investigated the effect of four abiotic stresses (ABA treatment, drought, cold and salt) on the expression pattern of splice variants by qRT-PCR. We selected Ws as the preferred ecotype for treatment since the previous study on *CIPK3* was also performed on the same ecotype, and in our study Ws had the highest cumulative expression of the splice variants (Kim et al., 2003, **Figure 1B**). ABA and drought were able to induce the expression of the entire CIPK3 splice variant pool to significantly higher levels (**Figure 3**). Compared to the former two stresses in cold and salt stress, the transcript levels of the *CIPK3* splice variants were lower. The *CIPK3.1* splice variant had the highest transcript level in all the conditions and was followed by *CIPK3.4*, which had the second highest transcript abundance. The presence of *CIPK3.2*, *CIPK3.3* and *CIPK3.5* were much lower than both *CIPK3.1* and *CIPK3.4*. However, both ABA and drought stress were able to significantly enhance the induction of all the CIPK3 splice variants.

We also analyzed the expression profile of the splice variants after challenging them with *Pseudomonas syringae*, Pst (DC3000). For this experiment Col-0 was used to get rid of ecotype bias. CIPK3 AS variants showed induction in response to the pathogen challenge, and again *CIPK3.1* and *CIPK3.4* showed higher transcript abundance in comparison to *CIPK3.2*, *CIPK3.3* and *CIPK3.5*.

### ABR1 Interact with CIPK3.1 and CIPK3.2 But Not with CIPK3.3 and CIPK3.4

**FIGURE 4:**
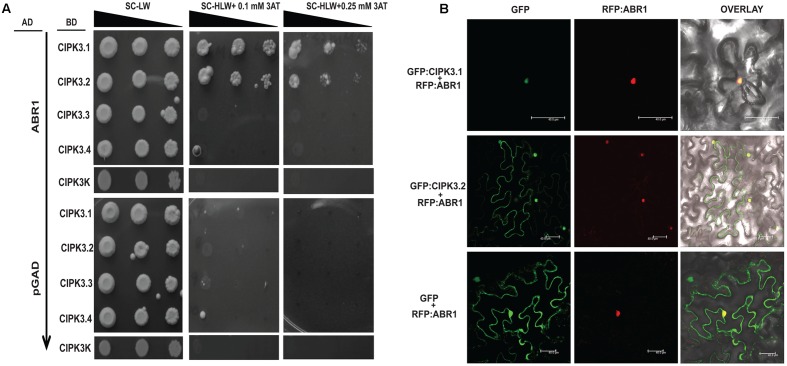
ABR1 physically interacts with only CIPK3.1 and CIPK3.2. **(A)** Dilution series of yeast AH109 strains transformed with AD-ABR1 and BD-CIPK3.1, BD-CIPK3.2, BD-CIPK3.3 and BD-CIPK3.4. Yeast two-hybrid analysis identified ABR1 as a common interactor of CIPK3.1 and CIPK3.2 interactor. The decreasing cell densities in the dilution series are illustrated by narrowing triangles. Yeast was grown on SD-LW medium (first column), SD-HLW + 0.1 mM 3AT medium (second column) and SD-HLW + 0.25 mM 3AT medium (third column). Results of negative control show no autoactivation of any variant. **(B)** Co-localization of ABR1 with CIPK3.1 and CIPK3.2 proteins in the epidermal peel cells of *Nicotiana benthamiana*. The upper panel depicts the co-expression of GFP:CIPK3.1 with RFP:ABR1 and display the formation of CIPK3-ABR1 complex in the nuclei. In the middle panel the same phenomenon is observed with CIPK3.2 and ABR1. The cell where RFP:ABR1 is not expressed, GFP:CIPK3.2 is seen in the cytoplasm. As negative control RFP:ABR1 is expressed with only GFP in the lower panel.

Our previous study has shown than CIPK3 interacts with an AP2-domain containing transcription factor, ABR1 and both protein accumulate in nucleus when co-expressed in *N. benthamiana* (Sanyal et al., 2017). We further performed Y2H to analyze the interaction of ABR1 with the splice variants of CIPK3. The Y2H results showed that ABR1 interacted only with CIPK3.1 and CIPK3.2 but not with CIPK3.3 and CIPK3.4 (**Figure 4A**). To verify that the interaction was dependent on the C-terminal of the proteins, we also used CIPK3K, which represent the kinase domain (without regulatory domain) of all splice variants. And we found that there was no interaction with the CIPK3K suggesting that the interaction is dependent on the C-terminal (regulatory domain) and the tail region of the splice variants. This was also verified using the ONPG based β- galactosidase assay (**Supplementary Figure S3B**). Both results indicate that the ABR1 seems to prefer CIPK3.1 showing strongest interaction with it followed by CIPK3.2 (**Figure 4A**). The co-expression of both the GFP:CIPK3.1 and GFP:CIPK3.2 in *N. benthamiana* epidermal peel cell led to the accumulation into the nucleus along with RFP:ABR1. This indicates that these protein complexes might be functional inside the nucleus *in planta* (**Figure 4B**).

## Discussion

The CBL-CIPK sensor responder pair has been reported in plants as one of the Ca^2+^ mediated stress sensors (Luan, 2009; Kudla et al., 2010; Zhu, 2016). Evolutionarily, the Arabidopsis CIPKs can be divided into an intron rich group and an intron poor group (Kolukisaoglu et al., 2004). The presence of the introns naturally makes these CIPKs target of AS and generation of splice variants. In Arabidopsis there are five CBLs [*CBL1* (2 variants), *CBL3* (2 variants), *CBL4* (2 variants), *CBL9* (2 variants) and *CBL10* (3 variants)] and four CIPKs [*CIPK1* (2 variants), *CIPK3* (5 variants), *CIPK8* (2 variants), *CIPK9* (3 variants)] that generate alternatively spliced transcripts. The *CIPK3* gene possesses the highest number of splice variants and hence it caught our attention to explore and became an interesting target for analysis. Moreover, the investigation of *CIPK3* was also important since it has 4 orthologs in the Oryza genome, and TIGR database search show three of them [*OsCIPK31* (8 variants), *OsCIPK32* (3 variants) and *OsCIPK33* (2 variants)] generate splice variants (Kanwar et al., 2014).

Alternative splicing usually generates a full-length protein followed by other truncated proteins due to the presence of premature stop codon (Lin et al., 2010; Wang et al., 2015). The sequence analysis of all the spliced variants from our sequenced data and TAIR database provided us with the information that none of the variants had any truncation of important/defined CIPK motifs. Probably as a result, the splice variants retain their ability to localize and choose similar upstream regulators and phosphorylate them. CBL2 and CBL3 in the recent years have been proved as interactors of CIPK3 (Tang et al., 2015). The fact that they interacted with all the tested splice variants and showed a stronger interaction (β-gal quantification assay) indicated that they might be the master regulators of CIPK3. In earlier analysis, CBL9 has been identified as the one of the major upstream target of CIPK3 *in vitro* and *in vivo* (Pandey et al., 2008; Sanyal et al., 2017). Recently, an interesting report from the Luan laboratory has provided us important clues about the “controllers” of CIPK3, which are more diverse than previously imagined (Tang et al., 2015). We believe, to the best of our knowledge, this is the first attempt to find out all the upstream regulators (read CBL) of CIPK3. The growth based Y2H assay and quantitative measurement of β-gal results point that four CBLs, i.e., CBL1, CBL2, CBL3 and CBL9 interact with CIPK3. Most of them actively participate in transducing stress signals during drought and ABA beside other stresses and development (Albrecht et al., 2003; Cheong et al., 2003, 2007; Pandey et al., 2004; Batistic et al., 2012). The fact that CBL6 show a specific interaction pattern with only CIPK3.3 and CIPK3.4 is an interesting observation. In the regulatory region of CIPK, the region after the PPI domain is yet to be characterized (Gong et al., 2004). The paradigm of CBL-CIPK interaction emphasizes that NAF/FISL determine the interaction of CBL with CIPK (Halfter et al., 2000; Albrecht et al., 2001; Guo et al., 2001). At the molecular level, the hydrophobic residues of the NAF domain in the CIPK help it to bind to the residues in the hydrophobic crevice of the CBL (Sanchez-Barrena et al., 2007). Interestingly, the difference in the splice variants starts after the PPI motif, and in the light of the present data, it seems quite possible that the interaction is dependent on some “yet unidentified” aspect that involves the C-terminal tail, which is a uncharacterized region in CIPK’s (Gong et al., 2004).

The detection of the spliced transcripts during unstressed and stressed conditions indicate that CIPK3 undergoes AS in both conditions, but the higher transcript abundance observed under stress indicates that it is a stress responsive gene corroborating the earlier report on CIPK3 (Kim et al., 2003). The comparatively higher expression in ABA and drought further strengthens CIPK3’s role in ABA signaling (Pandey et al., 2008; Sanyal et al., 2017). *CIPK3.5* is the least expressed among the other variants of CIPK3 (**Figures 1B**, **3**) and probably for this reason; we were unable to amplify a cDNA and consequently failed to explore it at protein level in our study. But examining the 57 reported CIPK3 ESTs from NCBI database, we found one particular EST (AI995848), which shows more similarity to CIPK3.5 than others. So we hypothesize that expression of *CIPK3.5* might be related to a very specific condition, which still need to be investigated. The splice variants displayed another major difference in their regulation in choosing one of the downstream interactors, ABR1. Only CIPK3.1 and CIPK3.2 were able to interact with ABR1 while CIPK3.3 and CIPK3.4 variants did not show any preference for ABR1. This could be the possible reason why Pandey and colleagues failed to deduce a physical interaction of ABR1 and CIPK3 in their study because they probably used the CIPK3.3 variant in their study (Pandey et al., 2005). The interaction pattern indicated that the motif for ABR1 binding/recognition could be the unique amino acids at the end. Here we are not sure at this point how different tail of CIPK3.1 and CIPK3.2 enable interaction with ABR1. We hypothesize that these amino acids at the tail form some sort of a hook at CIPK3.1 and CIPK3.2’s C-terminus, which probably allow efficient binding with ABR1 in Y2H assays. In the two other splice variants that we have tested, the tails are larger and probably the hook is too long to bind efficiently with ABR1. This could also be true for CIPK3.5 as well, as this particular splice variant has a longer tail in comparison to CIPK3.1 and CIPK3.2. No interaction of CIPK3K variant probably infers that along with the tail the actual binding region was also cut-off from this protein. These results, however, definitely clarifies that the interaction with ABR1 is not mediated by the N-terminal (catalytic kinase domain) of CIPK3.

**FIGURE 5:**
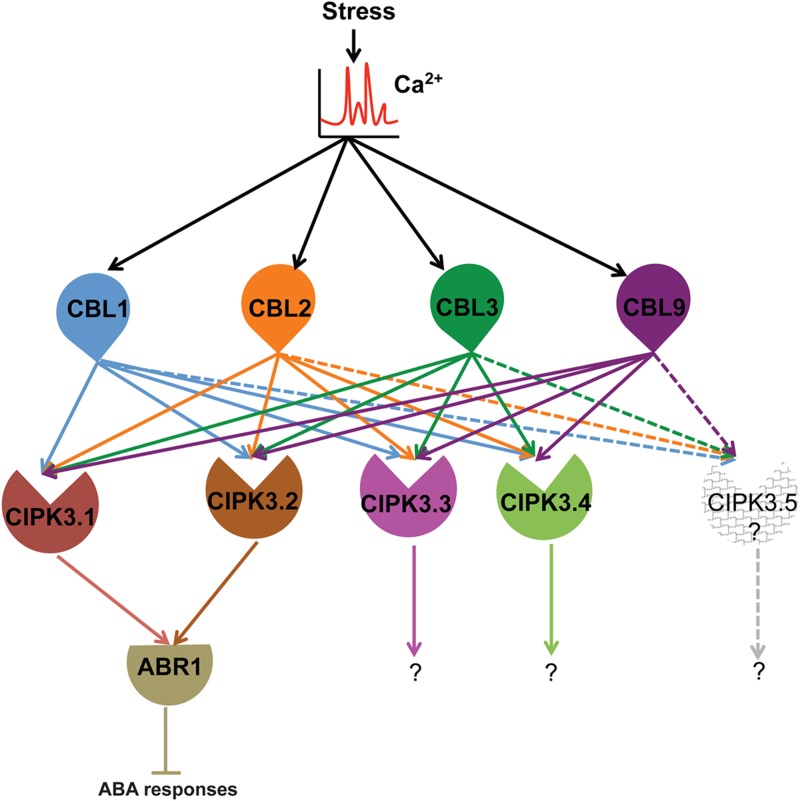
Hypothetical model depicting the functional role of CBLs-CIPK3 splice variants and downstream targets in stress signaling in *Arabidopsis.* We suggest Ca^2+^ signal is sensed by CBLs and the splice variant are activated depending on their availability. Stress signals (abiotic and high magnesium) lead to the generation of Ca^2+^ signature. The elevated Ca^2+^ level is sensed by four CBLs (CBL1, 2, 3, and 9). Specifically during ABA/drought signaling CBL1, CBL2 and CBL9 can activate CIPK3.1 and CIPK3.2 and they in turn are able to interact with ABR1 and regulate its function probably by phosphorylation and negatively regulating ABA signaling. The other variants although are activated by the same set of CBLs probably choose distinctly different targets to complete the signal transduction event. The other CBL-CIPK3 splice variant-target combination may similarly work in different physiological signal transduction pathways. The existence of CIPK3.5 is a matter of debate as we could not amplify the full-length transcript from Arabidopsis and hence we leave it as an open question for future research.

Summarizing the present knowledge on CIPK3 and this study, we put forward this hypothesis for the *in-planta* functioning of CIPK3 splice variants (**Figure 5**). Ca^2+^ signals generated by abiotic stresses (ABA, drought, cold and salt) and during magnesium (Mg^2+^) homeostasis results in the CBL mediated activation of CIPK3 (Kim et al., 2003; Pandey et al., 2008; Tang et al., 2015). During Mg^2+^ homeostasis, CBL2/CBL3 duo controls the quartet CIPK3/9/23/26 to sequester Mg^2+^ to tonoplast (Tang et al., 2015). The same quartet by phosphorylating SnRK2D/E/I can also help in Mg^2+^ sequestration. (Mogami et al., 2015; Tang et al., 2015). However, during ABA responses, plants use discrete CIPK mediated signaling pathways (Song et al., 2005; Lyzenga et al., 2013; Zhou et al., 2015; Sanyal et al., 2017). Amongst the multiple CIPK pathways, in the CIPK3-ABR1 pathway, ABR1 might be controlled by two splice variants CIPK3.1 and CIPK3.2. In other reports, ABI5 has been shown to be controlled by two different CIPKs, CIPK11 and CIPK26 during ABA signaling (Lyzenga et al., 2013; Zhou et al., 2015). So we assume that a similar kind of control is manifested on ABR1 by the two splice variants of CIPK3 during ABA signaling. Regarding the upstream regulator of CIPK3, at least two of the CBLs (CBL2 and CBL9) have been proven role in ABA signaling and CBL1 is involved in drought stress (Albrecht et al., 2003; Cheong et al., 2003; Pandey et al., 2004; Batistic et al., 2012). Also based on the position of canonical amino acid residues in the EF-hand sequence of CBLs (Sanyal et al., 2016), we may hypothesize that the Ca^2+^ binding affinity might be in the order of CBL9 first, then CBL1 and CBL2 and lastly CBL3. Hence these CBLs (CBL1, CBL2, CBL3 and CBL9) might serve to keep CIPK3 variants active at different levels of Ca^2+^ present in the cell. After being activated by the CBLs, CIPK3.1 and CIPK3.2 take the separate ABR1 mediated pathway to propagate the signaling event. The CIPK3.3 and CIPK3.4 variants, on the other hand, probably choose some other targets than ABR1 to advance the Ca^2+^ signaling through a very different pathway and depending on their similar protein structure they may choose a similar target for modulation. CIPK3.5 that is distinctly different from the other four might altogether choose a very different target for propagating the Ca^2+^ signals.

## Author Contributions

GKP conceived the project; SKS, PK, HS, KK, and SKJ performed experiments; SKS and GKP analyzed data and wrote the manuscript.

## Conflict of Interest Statement

The authors declare that the research was conducted in the absence of any commercial or financial relationships that could be construed as a potential conflict of interest.

## Footnotes

1 www.arabidopsis.org

2 www.ncbi.nlm.nih.gov/IEB/Research/Acembly/

3 http://www.cbs.dtu.dk/services/TargetP/

4 https://wolfpsort.hgc.jp/

## Supplementary Material

**FIGURE S1 |** Alignment of amino acids and motifs of CIPK3 splice variants. The amino acid sequences of all five-splice variants were taken from TAIR and sequence alignment was performed using DNASTAR. The major difference originates at the C-terminus and only CIPK3.4 has different and extended N-terminal. The typical kinase domain (along with activation segment and conserved residues), NAF/FISL motif and PPI domain have been marked in the figure with different colored boxes.

**FIGURE S2 |** Alignments of cDNA sequence of CIPK3 splice variants for selection of the segments to be analyzed in qRT-PCR and the actual amplification of the amplicons and analysis of their sizes by agarose gel electrophoresis.

**FIGURE S3 |** Negative control of the yeast two-hybrid experiment and β-gal assay CIPK3.1 and CIPK3.2 with ABR1. **(A)** None of the CBLs tested for Y2H assay show transactivation. Dilution series of yeast AH109 strains co-transformed with pGAD.GH-(Empty) and BD-(CBL1 to CBL10). The decreasing cell densities in the dilution series are illustrated by narrowing triangles. Yeast was grown on SD-LW medium (first column), SD-HLW + 0.5 mM 3AT (second column) and SD-HLW + 3 mM 3AT (third column). **(B)** Quantitative analysis of β-galactosidase activity of yeast colonies containing ABR1-CIPK3.1 and ABR1-CIPK3.2. Data shown are in Miller Units.
