# Supplementary figures and images for: Alternative Splicing of CIPK3 Results in Distinct Target Selection to Propagate ABA Signaling in Arabidopsis
Source: Front Plant Sci. 2017 Nov 24;8:1924. doi: 10.3389/fpls.2017.01924 (PMC5705611; doi:10.3389/fpls.2017.01924)

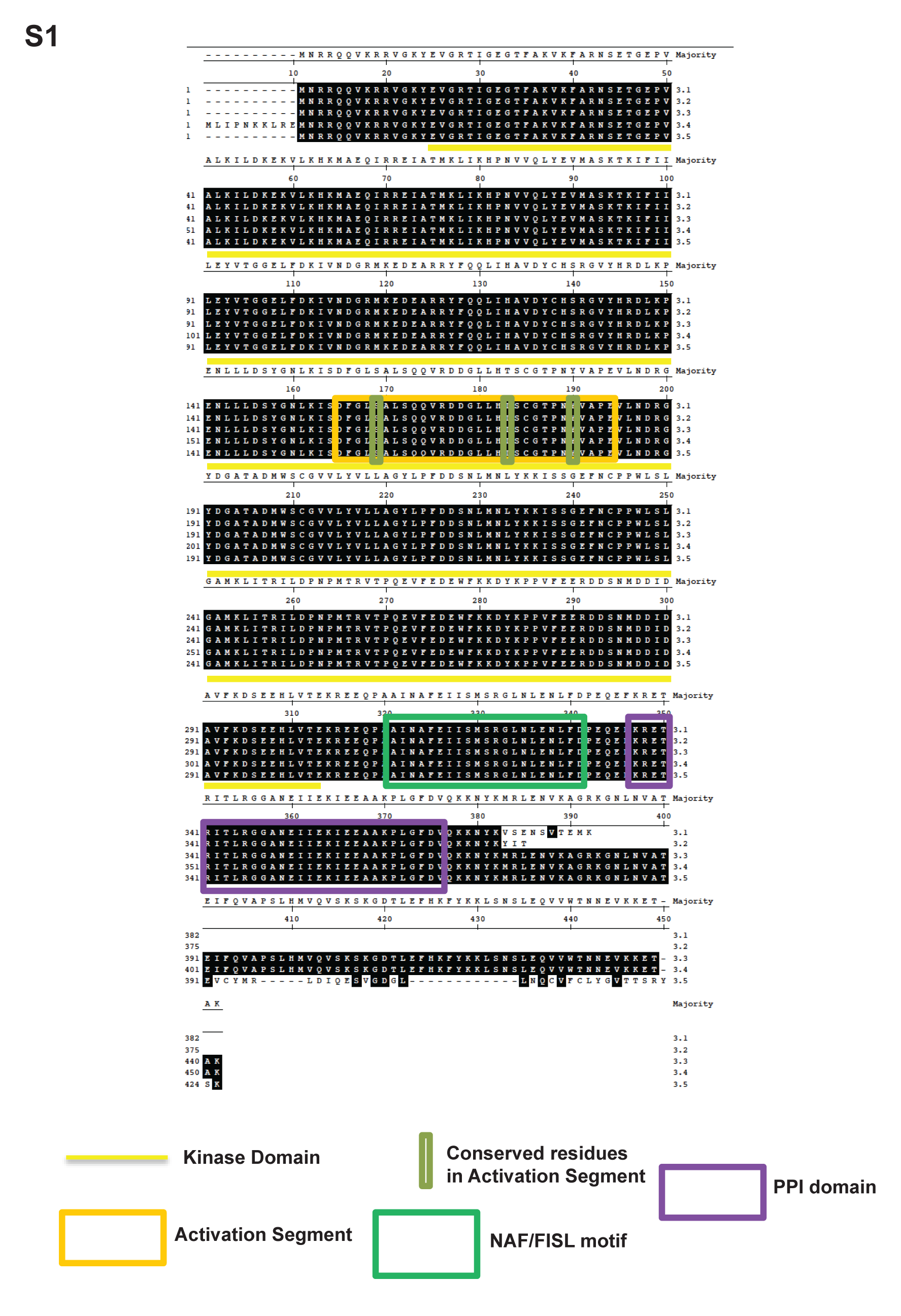

Supplement: Supplementary file 1 [file Image_1.TIF]

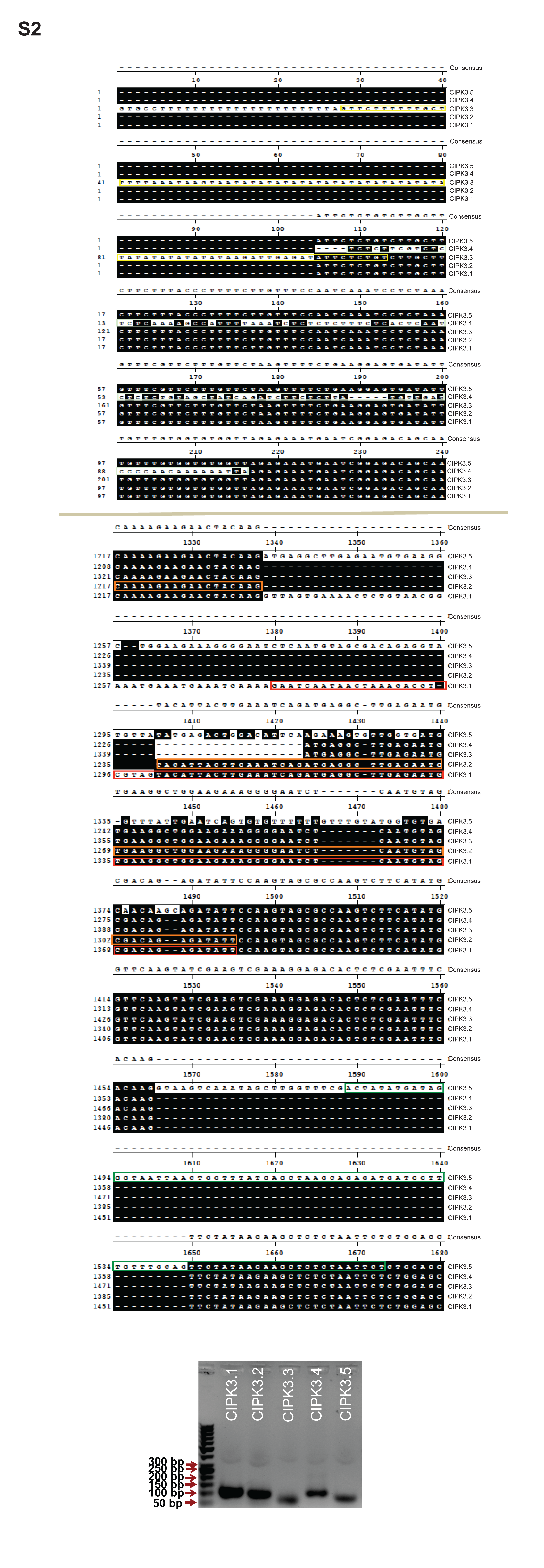

Supplement: Supplementary file 2 [file Image_2.tif]

S3A

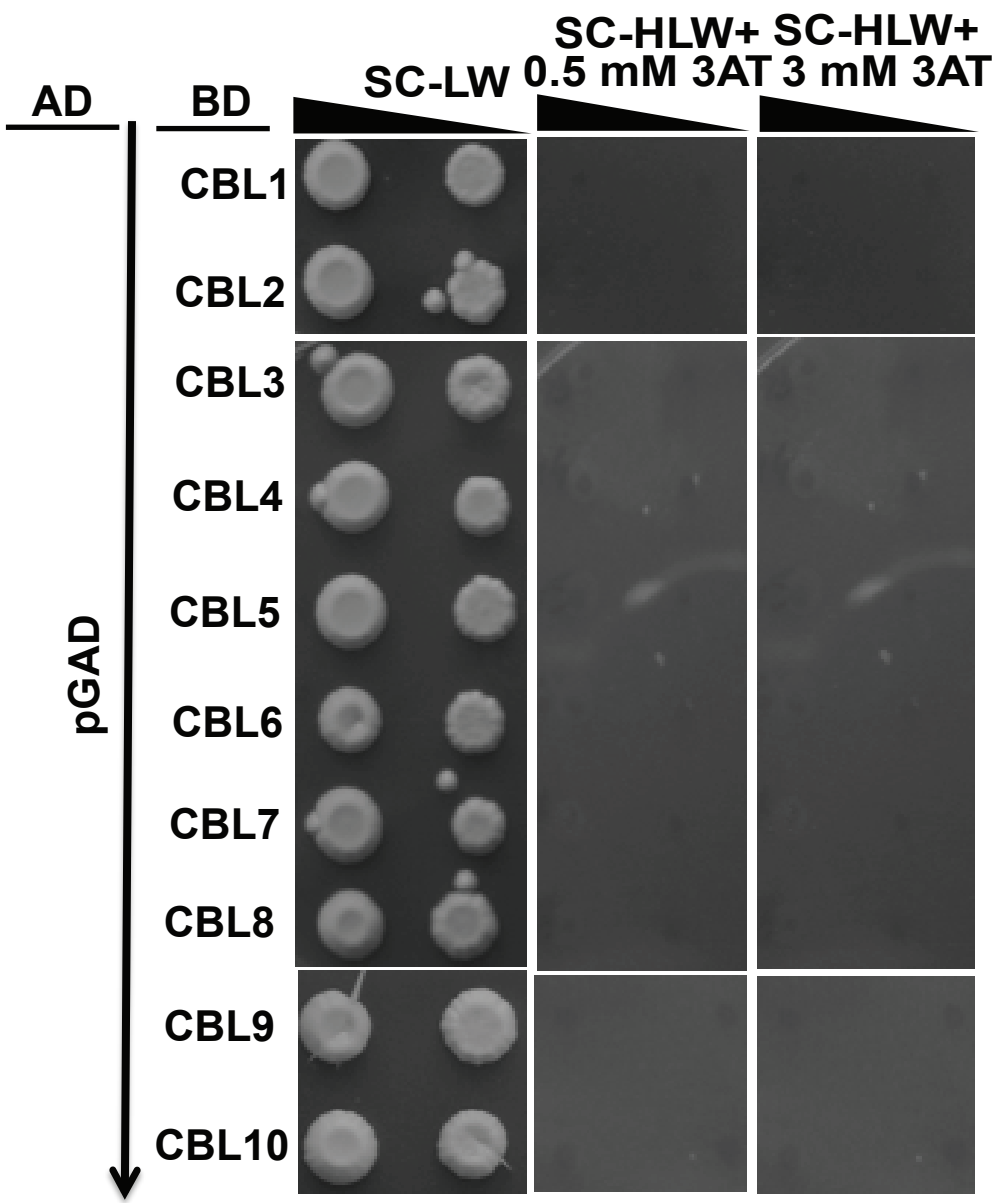

S3B

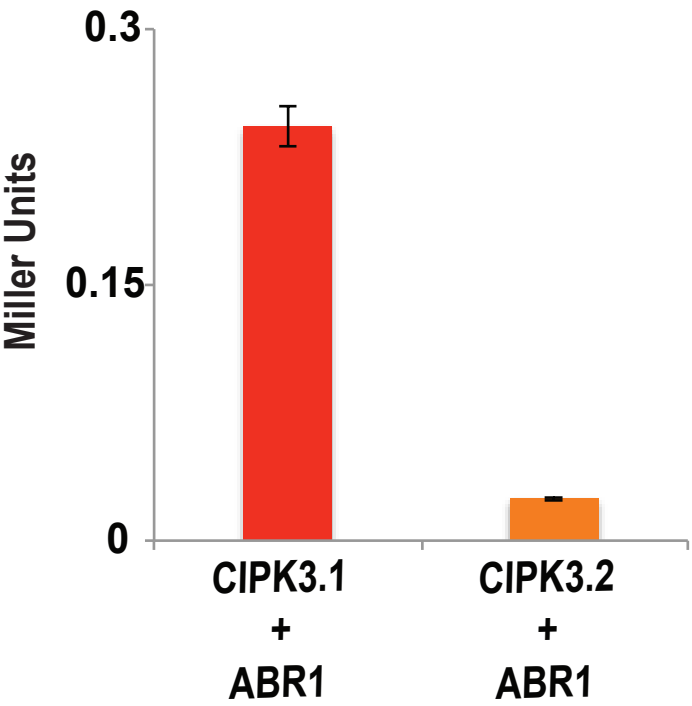

Supplement: Supplementary file 3 [file Image_3.pdf]
