# Supplementary material for: Alternative Splicing of CIPK3 Results in Distinct Target Selection to Propagate ABA Signaling in Arabidopsis
Source: Front Plant Sci. 2017 Nov 24;8:1924. doi: 10.3389/fpls.2017.01924 (PMC5705611; doi:10.3389/fpls.2017.01924)
Supplement: Supplementary file 4 [file Table_1.pdf]

Supplementary Table1. List of clones and publications of CIPK3 splice variants

Splice variant	NCBI Clone id	Published references
CIPK3.1	AF367290<br>AY059163	(Kolukisaoglu et al., 2004; Sanyal et al., 2017)
CIPK3.2	AF286051	(Kolukisaoglu et al., 2004; Batistic et al., 2010)
CIPK3.3	AY091098<br>AY142671<br>AY266298	(Kim et al., 2000; Kolukisaoglu et al., 2004; Pandey et al., 2008)
CIPK3.4	Not available	(Gao et al., 2012)
CIPK3.5	Not available	Not available
