# Supplementary material for: Alternative Splicing of CIPK3 Results in Distinct Target Selection to Propagate ABA Signaling in Arabidopsis
Source: Front Plant Sci. 2017 Nov 24;8:1924. doi: 10.3389/fpls.2017.01924 (PMC5705611; doi:10.3389/fpls.2017.01924)
Supplement: Supplementary file 5 [file Table_2.pdf]

Supplementary Table 2. List of primers used for qRT-PCR

Splice Variant Name	Primer Name	Sequence 5' → 3'	Amplicon spread in cDNA	Lenth of Amplicon
**CIPK3.1**	CIPK3.1RT F	GAATCAATAACTAAAGACGTCGT	1276 to 1380	104 bp
	CIPK3.1RT R	AATATCTCTGTCGCTACATTGAGA		
**CIPK3.2**	CIPK3.2RT F	CAAAAGAAGAACTACAAGTACA	1217 to 1314	97 bp
	CIPK3.2RT R	AATATCTCTGTCGCTACATTGA		
**CIPK3.3**	CIPK3.3RT F	AGTTCTTTTTTGCTTTTTAAATAAG	28 to 113	85 bp
	CIPK3.3RT R	CAGAGAATATCTCAATCTTATATAT		
**CIPK3.4**	CIPK3.4RT F	TCTCTTCGTCTCTCTCAAAA	1 to 103	103 bp
	CIPK3.4RT R	TAATTTTTTGTTGGGGATCAACA		
**CIPK3.5**	CIPK3.5RT F	ACTATATGATAGGGTAATTAAC	1482 to 1566	84 bp
	CIPK3.5RT R	AGAATTAGAGAGCTTCTTATAG		
