# Supplementary material for: Alternative Splicing of CIPK3 Results in Distinct Target Selection to Propagate ABA Signaling in Arabidopsis
Source: Front Plant Sci. 2017 Nov 24;8:1924. doi: 10.3389/fpls.2017.01924 (PMC5705611; doi:10.3389/fpls.2017.01924)
Supplement: Supplementary file 6 [file Table_3.pdf]

Supplementary Table 3. List of primers used for generation of constructs

Purpose	Constructs	Name	Sequence 5' → 3’
Y2H	CIPK3.1AD/BD	CK3.1 AD/BD F'BamHI	AGGATCCCATGAATCGGAGACAGCAA
		CK3.1 pGBT9 R Sal1	GTCGACTCATTTCATTTCCGTTACA
	CIPK3.2AD/BD	CK3.1 AD/BD F'BamHI	AGGATCCCATGAATCGGAGACAGCAA
		CK3.2 pGBT9 R Sal1	AGTCGACTCAAGTAATGTACTTGTAGT
	CIPK3.3AD/BD	CK3.1 AD/BD F'BamHI	AGGATCCCATGAATCGGAGACAGCAA
		CK3.4 pGBT9 R Sal1	GTCGACCTTCTCTCTTCTCAGTAAC
	CIPK3.4AD/BD	CK3.4 AD/BD F'BamHI	AGGATCCCATGTTGATCCCCAACAAA
		CK3.4 pGBT9 R Sal1	GTCGACCTTCTCTCTTCTCAGTAAC
	CIPK3KAD/BD	CK3.1 AD/BD F'BamHI	AGGATCCCATGAATCGGAGACAGCAA
		CK3K R Sal1	GTCGACCTCAGTAACAAGATGTTCCT
Localization	CIPK3.1TOPO	CK3.1 F	CACCATGAATCGGAGACAGCAAGT
		CK3.1 R	TTTCATTTCCGTTACAGAGT
	CIPK3.2TOPO	CK3.1 F	CACCATGAATCGGAGACAGCAAGT
		CK3.2 R	AGTAATGTACTTGTAGTTCT
	CIPK3.3TOPO	CK3.1 F	CACCATGAATCGGAGACAGCAAGT
		CK3.4 R	CTTTGCTGTTTCTTTCTTAA
	CIPK3.4TOPO	CK3.4 F	CACCATGTTGATCCCCAACAAAAA
		CK3.4 R	CTTTGCTGTTTCTTTCTTAA
Site directed Mutagenesis	CIPK3.1T/DpGEX and CIPK3.4T/DpGEX	CK3T/D For	AGGGATGATGGACTCTTGCATGACTCGTGT GGAACACCAAACTAC
		CK3T/D Rev	GTAGTTTGGTGTTCCACACGAGTCATGCAA GAGTCCATCATCCCT
Protein	CIPK3.1pET/pGEX	CK3.1 Expn F BamH1	AGGATCCATGAATCGGAGACAGCAA
		CK3.1 pGEX R Xho1	ACTCGAGTCATTTCATTTCCGTTACA
	CIPK3.4pET/pGEX	CK3.4 Expn F BamH1	AGGATCCATGTTGATCCCCAACAAA
		CK3.4 pGEX R Xho1	ACTCGAGTCACTTTGCTGTTTCTTTC

Restriction endonuclease sites are underlined. For mutagenesis primers, the changed bases are underlined.
